# Caspase-mediated pathways in retinal ganglion cell injury: a novel therapeutic target for glaucoma

**DOI:** 10.3389/fcell.2025.1586240

**Published:** 2025-04-30

**Authors:** Nisha Rajakrishna, Seok Ting Lim, Xiaomeng Wang, Tina T. Wong

**Affiliations:** ^1^ Drug Delivery and Ocular Therapeutics, Singapore Eye Research Institute, Singapore, Singapore; ^2^ Centre for Vision Research, Duke-NUS Medical School, Singapore, Singapore; ^3^ Institute of Molecular and Cell Biology, A*STAR, Singapore, Singapore; ^4^ Glaucoma Department, Singapore National Eye Centre, Singapore, Singapore

**Keywords:** caspase, retinal cell death, glaucoma, neuroprotection, retinal ganglion cell, optic nerve, peptidomimetic inhibitor

## Abstract

Glaucoma is a complex disease of the optic nerve leading to vision loss and blindness, with high worldwide incidence and disproportionate prevalence in older populations. Primary open-angle glaucoma, caused by a reduction in outflow of aqueous humor through the trabecular meshwork, is the most common subset of the disease, though its underlying molecular mechanisms are not well understood. While increased intraocular pressure is the most common risk factor in glaucoma progression, the disease is ultimately characterized by the loss of retinal ganglion cells (RGCs) and destruction of the optic nerve. Given the irreversibility of RGC death, neuroprotection of RGCs is a promising avenue of glaucoma prevention and treatment. The caspase family of proteins are integral members of the apoptotic death cascade. They have been shown to play a significant role in RGC death in numerous models of retinal injury. Direct inhibition of several caspase family members, through targeted siRNAs and peptidomimetics, demonstrate promising capacity to reduce caspase expression and preserve RGCs following intraocular pressure increase or optic injury. A wide variety of alternative therapeutics targeted for RGC survival, including neurotrophins, immunomodulators, cytoprotectants, and endogenous hormones, also display indirect caspase-inhibiting capabilities. Following intraocular pressure increase or external retinal injury, both direct and indirect caspase inhibitors elicit higher RGC counts, increased RGC layer thickness, and attenuation of RGC damage, clearly demonstrating the neuroprotective abilities of caspase inhibitors. Caspase inhibition, particularly by direct approaches of siRNA or peptidomimetic-based therapeutics, has the potential to achieve substantial neuroprotection in the glaucomatous eye.

## Introduction

### Glaucoma

Glaucoma is a multifaceted optic neuropathy that is a leading cause of blindness across the globe. 76 million people worldwide had been diagnosed with glaucoma in 2020, with a projected rise to 111.8 million people by 2040 (Tham et al., 2014). Glaucoma disproportionately affects older populations, with 3.54% prevalence in patients aged 40–80 years. Other demographic groups, particularly males and those with African ancestry, also demonstrate a heightened likelihood of glaucoma (Tham et al., 2014; Allison et al., 2020).

Glaucoma is characterized by the destruction of retinal ganglion cells (RGCs), degeneration of the optic nerve, and thinning of the retinal nerve fiber layer (Schuster et al., 2020). The disease is fundamentally considered an axonopathy due to its degradative pathology, affecting the distal axon in a Wallerian nature (Vishwaraj et al., 2022). In patients, this manifests as progressive visual field loss, especially in peripheral regions, and a decrease in vision quality. Glaucoma cases can be classified into two subcategories–angle-closure and open-angle–both of which are often accompanied by a rise in intraocular pressure (IOP), a known risk factor. In acute angle-closure glaucoma, the chamber angle lying between the peripheral posterior corneal surface and iris is occluded by the iris itself (Schuster et al., 2020). This results in the sudden blockage of aqueous humor outflow, presenting as a rapid onset of ocular or cranial pain, blurred vision, and changes to the visual field (Khazaeni et al., 2025). In contrast, open-angle glaucoma results from an increased resistance of aqueous humor outflow through the trabecular meshwork, despite the presence of a structurally open drainage angle (Morgan and Yu, 2019). It is important to note that open-angle glaucoma is often but not always accompanied by elevated IOP. Irrespective of the type of glaucoma, the intraocular buildup of fluid damages RGCs, the retinal nerve fiber layer, and the optic nerve itself. Optic disc distortion is another feature common to the glaucomatous eye, caused by the retroactive curving of the underlying lamina cribrosa connective tissue. An empty space develops and enlarges in the optic disc’s center, followed by the deepening of the disc’s curvature; these two features are known as “cupping” and “excavation” respectively (Greco et al., 2016). These elements result in a higher cup-to-disc (C:D) ratio, another hallmark characteristic of glaucoma.

### RGC death and its mechanisms

Elevated IOP is the sole modifiable risk factor for glaucoma, and for many decades the principal objective of glaucoma treatment has been the reduction of IOP. A broad range of G-protein-coupled receptors (GPCRs) have been revealed as targets of IOP-reducing agents, including β-adrenergic antagonists, α-adrenergic agonists, and prostaglandin analogs (He et al., 2018). These pharmaceutical therapeutics continue to be the first-line drugs for glaucoma patients with elevated IOP, as denoted by the American Academy of Ophthalmology guidelines (Gedde et al., 2021). However, sustained optic nerve and RGC damage despite the reduction of IOP was demonstrated in several trials, including the Early Manifest Glaucoma Trial and the Collaborative Initial Glaucoma Treatment Study (Lichter et al., 2001; Heijl et al., 2002). Similarly, a lack of glaucomatous optic neuropathy was exhibited in cases of IOP far above the standard threshold, as seen in the Ocular Hypertension Study (Guymer et al., 2019; Kass et al., 2002). It is thus hypothesized that while IOP is undoubtedly an important factor contributing to ocular nerve degeneration, there must exist other pathologic mechanisms behind the progressive optic neuropathy in glaucoma and accordingly, alternative methods of treatment.

Considering the contribution of RGC and optic nerve destruction to glaucoma progression, and the inability of RGCs to regenerate, strategies to promote RGC survival could likely offer a pervasive defense to the glaucomatous eye. Based on experimental models of glaucoma and optic nerve injury, there are several key pathways involved with RGC death. Neurotrophic factors expressed in the brain and transported to the retina provide a universal neuroprotective effect and are key players in the maintenance of healthy retinal cells. Brain-derived neurotrophic factor (BDNF), a neurotrophic factor affecting neuronal growth and plasticity *via* interaction with Tropomyosin receptor kinase B (TrkB) and P75 neurotrophic receptor (p75NTR), preserves retinal integrity throughout both the conventional aging process and external injury. BDNF is also directly produced by the ganglion cell layer and inner nuclear layer within the retina. Upregulation of BDNF is observed in both experimental glaucoma cases and optic nerve axotomy experiments (Gupta et al., 2014; Ko et al., 2000), and BDNF has proven neuroprotective effects through promoting the survival and development of RGCs *in vitro* in several studies (Johnson et al., 1986; Watanabe and Fukuda, 2002). Ciliary neurotrophic factor (CNTF), a cytoplasmic protein known for neuronal differentiation by activating the CNTFR-α receptor, exerts a similar effect on retinal cells. Expression of CNTF increases following optic nerve axotomy as well as in ischemic and glaucomatous eye cases (Weise et al., 2000). Pigment epithelium-derived factor (PEDF), known for its neuroprotective and anti-angiogenic functions, reduces retinal ganglion cell death from optic nerve crush through a glial cell-mediated mechanism (Vigneswara and Ahmed, 2019). Glial cell line-derived neurotrophic factor (GNDF), yet another neuronal survival-promoting neurotrophic factor, was also shown to provide retinal neuroprotective effects immediately following optic nerve transection (Klöcker et al., 1997). Significantly, neurotrophic factors including BDNF, CNTF, PEDF, and GDNF, widely utilize retrograde axonal transport for movement between the brain and the RGC layer. Elevated IOP has been reported to impede both anterograde and retrograde axonal transport (Watanabe and Fukuda, 2002), which may cause RGC degeneration by restricting neurotrophin entry into the eye, therefore leading to the development of ocular diseases. There is thus a direct pathway linking IOP elevation, neurotrophic factor reduction, and optic neuropathy.

While IOP elevation and neurotrophic factor reduction are upstream instigators of optic degeneration, the direct process responsible for the loss of RGCs is apoptosis (Kerrigan et al., 1997). Neurotrophic factors including BDNF, CNTF, and GDNF achieve neuroprotection through regulating the phosphatidylinositol 3-kinase (PI3K)/protein kinase B (Akt) and mitogen-activated protein kinase (MAPK)/extracellular signal-regulated kinase (ERK) signaling pathways (Levkovitch-Verbin, 2015). Deprivation of these factors obstructs this vital axis for RGC survival, leading to an imbalance between pro-survival and pro-apoptotic pathways and an ultimate upsurge in apoptotic signaling. MAPKs, a group of protein kinases especially fundamental to apoptosis, consist of three family members: ERKs, c-Jun N-terminal kinases (JNKs), and p38 kinases. All three varieties of MAPKs participate in the cell death of RGCs. Pro-survival kinases ERK1 and ERK2 are upregulated through neurotrophic regulation to promote RGC survival (Almasieh et al., 2011). Inhibitors of pro-apoptosis kinase JNK have provided additional neuroprotection in several hypertension models (Liu et al., 2011). Pro-apoptotic kinase p38 has demonstrated increased activation in induced RGC injury models, indicating its role in optic neuropathy pathways (Levkovitch-Verbin et al., 2007). As apoptosis is the prominent mechanism behind RGC death, furthering our understanding of key proteins in the apoptotic signaling cascade could provide future targets for neuroprotection.

## Existing neuroprotective agents and challenges

Neuroprotection and the preservation of RGCs have been newly established as promising methods of glaucoma prevention and treatment. Four criteria have been proposed to assess the neuroprotective potential of pharmaceutical agents (Vishwaraj et al., 2022). A potential candidate should 1) have target receptors in the retina or optic nerve; 2) demonstrate neuroprotective effects in animal studies through significant improvement to RGC survival; 3) reach neuroprotective concentrations in the posterior segment after clinical dosing; and 4) indicate neuroprotection in clinical trials.

There currently exist several neuroprotective approaches that meet multiple of these criteria, but no single pharmaceutical agent to date satisfies all four metrics. The strategies and targets that underlie the current approaches to neuroprotection are widespread and varied. Some IOP-reducing agents, especially α-2 adrenergic agonists and prostaglandin analogs, have demonstrated neuroprotective effects independent of their IOP-reducing mechanisms. Most notably, brimonidine tartrate is an α-2 adrenergic agonist that induces retinal vasodilation and manages aqueous humor movement (Wheeler et al., 2003). It has demonstrated the ability to promote RGC survival in several laboratory optic injury models of glaucoma. However, the neuroprotective mechanisms of this pharmaceutical and other IOP-reducing agents are not fully understood. More research and clinical trials are required to elucidate and confirm their RGC-sustaining mechanisms independent of their effect on IOP. Alternative potential avenues of neuroprotection include a variety of inhibitors (i.e., caspase, carbonic anhydrase, NMDA receptors, rho-kinase, and tumor necrosis factor), antioxidants, vitamins, vasodilators, and stimulation of sigma receptors (Vishwaraj et al., 2022; He et al., 2018).

## Caspases

**FIGURE 1:**
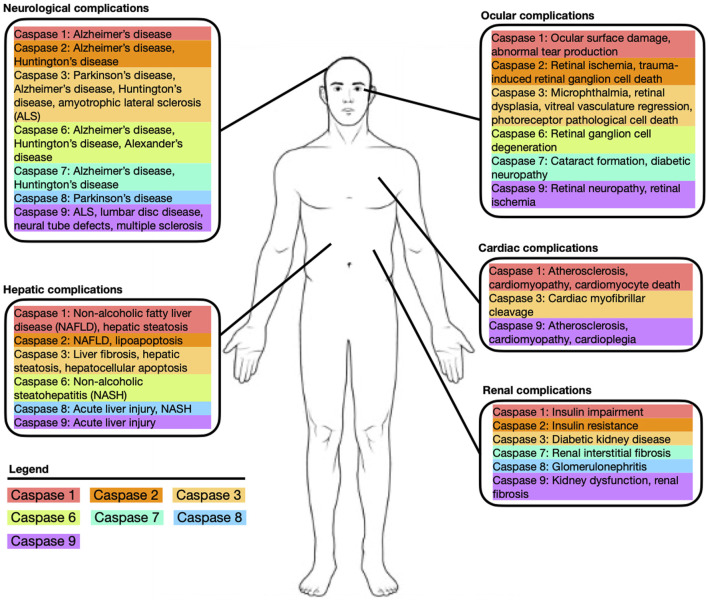
Depiction of common systemic diseases and complications associated with caspase-1, -2, -3, -6, -7, -8, -9.

Caspases are a class of cysteine proteases specific for cleavage after Asp residues (Nicholson and Thornberry, 1997) that control the apoptotic signaling cascade. They can be classified into inflammatory or apoptotic caspases, with the former category consisting of caspase–1, -4, and -5. Apoptotic caspases can be further classified into initiator (caspase–2, -8, -9, -10) and effector (caspase −3, −6, −7) caspases (McLuskey and Mottram, 2015). Caspases are implicated in the pathophysiology of numerous systemic disorders (Figure 1). Initiator caspases catalytically cleave the activation domain of executioner caspases to mobilize them. This commences a chain reaction of enzymatic cleavage and hydrolysis by activated executioner caspases, and related cascade proteins, to eventually induce apoptosis.

Caspases are initially produced as precursor pro-caspases which are constitutively expressed (Degterev et al., 2003). Pro-caspases can be cleaved quickly into the active form in response to specific, proximal cell signals within protein complexes that initiate the enzymatic apoptotic cascade (Thomas et al., 2017). This can be achieved through both extrinsic and intrinsic pathways, both of which culminate in the same execution pathway leading to apoptosis.

Extrinsically, the apoptotic caspase pathway is activated by the binding of extracellular ligands, namely, tumor necrosis factor (TNF-α), Fas ligand (Fas-L), and TNF-related apoptosis-inducing ligand (TRAIL), to the extracellular domain of transmembrane receptors such as Fas/CD95 and TNF receptor (TNFR) (Thomas et al., 2017). Specific death ligand binding prompts the recruitment of adaptor proteins, such as Fas-associated protein with death domain (FADD), Tumor Necrosis Factor Receptor-1-Associated Death Domain (TRADD), or Daxx-containing Death Domain. The interaction between transmembrane receptors and their respective adaptor proteins prompts the formation of the death-induced signaling complex (DISC), which triggers the activation of the key extrinsic apoptosis initiator: caspase-8 (Jan and Chaudhry, 2019). Following recruitment from FADD, oligomerization of procaspase-8 catalyzes its activation through self-cleavage. The pro-domain functional region remains attached to the DISC, but the active caspase-8 domain detaches to induce the activation cascade that sequentially cleaves caspase −3, −6, and −7. Caspase-8 can directly cleave procaspase-3 for its activation and successive induction of the caspase cascade. Alternatively, caspase-8 can participate in a mitochondria-related pathway of caspase-3 activation, whereby caspase-8 cleavage of Bcl2 Interacting Protein (BID) triggers cytochrome-c release to simulate the intrinsic apoptotic pathway (Kuwana et al., 1998).

The intrinsic pathway is mediated by mitochondria and predominantly activated by intracellular signals including DNA damage, oxidative stress, ER stress, and hypoxia (Jan and Chaudhry, 2019; Kuwana et al., 1998). Intracellular stress and resultant upregulation of p53 induces the insertion of Bax/Bak into the mitochondrial membrane, stimulating mitochondrial outer membrane permeabilization (MOMP). Pro-apoptotic factors, including cytochrome-c, second mitochondria-derived activator of caspase (Smac)/direct inhibitor of apoptosis-binding protein with low pI (DIABLO), and apoptotic protease activating factor 1 (Apaf-1) are subsequently released into the cytoplasm. The apoptosome, a wheel-shaped multi-protein complex, is produced upon the combination of cytochrome-c with Apaf-1 and subsequently recruits procaspase-9 to its domain (Elmore, 2007). The formation of the heptameric apoptosome triggers the self-cleavage of procaspase-9 and the subsequent cleavage and activation cascades of caspase-9, -3, and –7 respectively to complete proteolytic cleavage necessary for apoptosis.

## Role of caspases in neuroprotection

Seeing the importance of the caspase family in the apoptotic process--and the significant role of apoptosis in RGC death–it naturally follows that caspase proteins are highly involved in optic neurodegeneration. A variety of both initiator and effector caspases have roles in the degeneration of RGCs. Recent work has accordingly developed numerous caspase-based inhibitors with neuroprotective characteristics (Table 1).

**TABLE 1:** Summary of direct and indirect caspase inhibitors.

		Inhibitor	Caspase affected	Inhibition method	RGC damage method	Caspase inhibition pathway	Effect on RGCs	Study duration	References number
1		Melatonin	Caspase-1	Indirect	Ischemia-reperfusion injury	Inhibition via upregulation of NF-kB pathway	Reduction of RGC death from 71.9% to 39.1%	7 days	Zhang et al. (2021a)
2		GSK872, Nec-1	Caspase-1	Indirect	Glutamate-induced excitoxicity	Inhibition via suppression of RIP1/RIP3/MLKL pathway	Increased RGC density, retinal thickness, and GCC thickness compared to control	24 h	Liu et al. (2022)
3		YVAD-fmk	Caspase-1	Direct	NMDA (glutamate receptor agonist)	Direct inhibition	211 ± 31 cells/mm^2^ in treatment group compared to 316 ± 54 cells/mm^2^ in control group (**not effective)	31 h	Schuettauf et al. (2011)
4		siCASP2	Caspase-2	Direct	Optic nerve crush (ONC), optic nerve transection (ONT)	Direct inhibition	12-fold and 2.5-fold increase in RGC density for ONC and ONT respectively	7 days (ONC), 14 days ONT)	Ahmed et al. (2011)
5		v-VDVAD-fmk	Caspase-2	Direct	Optic nerve crush	Direct inhibition	60% increase in RGC survival compared to control	15 days	Vigneswara et al. (2012)
6		PEDF	Caspase-2	Direct	Optic nerve crush	Direct inhibition	63.2% increase in RGC survival compared to control	28 days	Vigneswara and Ahmed (2019)
7		PEDF-34	Caspase-2	Direct	Optic nerve crush	Direct inhibition	81.9% increase in RGC survival	28 days	Vigneswara and Ahmed (2019)
8		siCASP2	Caspase-2	Direct	Blunt eye injury	Direct inhibition	Increase in RGC survival immediately peripheral to the impact site (∼600um) compared to control	48 h	Thomas et al. (2018)
9		CaspNP	Caspase-3	Direct	Optic nerve crush	Direct inhibition	36.1% RGC loss in treated cells compared to 63.4% RGC loss in controls	48 h	Tawfik et al. (2021)
10		BDNF	Caspase-3	Direct	Optic nerve crush, optic nerve transection	Direct inhibition	Delayed RGC loss by 1 day compared to control	3, 5, 7 days	Sánchez-Migallón et al. (2016)
11		Z-DEVD_fmk	Caspase-3	Direct	Optic nerve crush, optic nerve transection	Direct inhibition	Delayed RGC loss by 1 day compared to control	3, 5, 7 days	Sánchez-Migallón et al. (2016)
12		Z-DEVD-FMK	Caspase-3	Direct	Fluid percussion injury	Direct inhibition	Consistently reduced RGC loss when injected at a variety of time points and concentrations compared to controls	21 days	Liu et al. (2015)
13		siCASP3	Caspase-3	Direct	Ischemia-reperfusion injury	Direct inhibition	Increased retinal thickness and RGC survival rate compared to control	2 days, 7 days	Ishikawa et al. (2012)
14		Latanoprost	Caspase-3	Indirect	Streptozotocin-induced diabetic retinal damage	p44/p42 MAPK-mediated inhibition	Reduction in TUNEL+ retinal neuroglial cells by 66.9% and 52.1% for 1M and 3M treatment compared to controls	5 days	Nakanishi et al. (2006)
15		Latanoprost	Caspase-3	Indirect	Serum deprivation, excessive glutamate, ONC	p44/p42 MAPK-mediated inhibition via FP receptors, MEK1/2-mediation inhibition	Increased RGC survival following serum deprivation and excessive glutamate damage methods compared to control	48 h	Kanamori et al. (2009)
16		DEVD-CHO	Caspase-3	Direct	Glutamate over-stimulation	Direct inhibition	Rescued 90% of total RGC population at peak concentration of 400 uM	5 days (40 min inhibitor treatment)	Chen et al. (2001)
17		Calpeptin (calpain-specific inhibitor)	Caspase-3	Indirect	Ionomycin and interferon-gamma exposure	Inhibition by suppression of upstream apoptotic activator calpain	Restoration of whole-cell membrane potential to baseline levels following calpeptin treatment	24 h	Banik et al. (2005)
18		DEVD-fmk	Caspase-3	Direct	NMDA (glutamate receptor agonist)	Direct inhibition	437 ± 77 cells/mm^2^in treatment group compared to 316 ± 54 cells/mm^2^in control group	31 h	Schuettauf et al. (2011)
19		z-DEVD-cmk	Caspase-3	Direct	Optic nerve transection	Direct inhibition	942 ± 99 cells/mm^2^ in treatment group compared to 379 ± 25 cells/mm^2^ in control group	14 days	Kermer et al. (1998)
20		DQMD-fmk	Caspase-3, caspase-6	Direct	NMDA (glutamate receptor agonist)	Direct inhibition	517 ± 78 cells/mm^2^ in treatment group compared to 316 ± 54 cells/mm^2^ in control group	31 h	Schuettauf et al. (2011)
21		Cyclosporine-A	Caspase-3, caspase-7	Indirect	Glutamate over-stimulation	ND	2.7-fold increase in cell viability and 2.5-fold increase in cell density compared to controls	24 h	Schultheiss et al. (2014)
22		Kaempferol	Caspase-3, caspase-8	Indirect	Ischemia-reperfusion injury	Inhibition via suppression of NLRP1/NLRP3 and NFkB/JNK pathways	Increased retinal thickness and RGC survival rate compared to control	48 h	Lin et al. (2019)
23		Resveratrol	Caspase-3, caspase-8	Indirect	Ischemia-reperfusion injury	Inhibition via suppression of Bax, HIF-1a/VEGF, and p38/p53 pathways	Increased inner retina thickness and RGC survival rate compared to control	7 days	Ji et al. (2021)
24		Morphine	Caspase-3, caspase-8	Indirect	Limbal venous hypertonic saline injection	Inhibition via TNFα suppression	Reduction in RGC loss for treatment group compared to controls	28 days	Husain et al. (2012)
25		IGF-I	Caspase-3, caspase-9	Direct	Optic nerve transection	Inhibition via upregulation of Pi3K/Akt antiapoptotic pathway	Increased RGC density as compared to controls	14 days	Kermer et al. (2000)
26		BDNF	Caspase-3, caspase-9	Direct	Optic nerve transection	Inhibition via upregulation of Pi3K/Akt antiapoptotic pathway	Increased RGC density as compared to controls	14 days	Kermer et al. (2000)
27		Ginkgo biloba	Caspase-3, caspase-9	Indirect	Hydrogen peroxide-induced oxidative damage	Inhibition via suppression of p53 acetylation and production of reactive oxygen species	87.5% RGC viability in treatment group compared to 43.6% for controls	48 h	Yu et al. (2022)
28		L-817,818 (somatostatin receptor 5 agonist)	Caspase-3, caspase-9	Indirect	Anterior chamber injection of superparamagnetic microspheres	Inhibition via elevation of Bcl-2, reduction of Ca^2+^, and suppression of AMPA receptors	1,317 RGCs per mm^2^at endpoint compared to 1,053 RGCs per mm^2^for controls	4 weeks	Zhang et al. (2021b)
29		Geranylgeranylacetone	Caspase-3, caspase-9	Indirect	GLAST +/- normal tension glaucoma model	Inhibition via suppression of HSP70	4978 RGCs per mm^2^ at endpoint compared to 4445 RGCs per mm^2^ for controls at highest treatment concentration of 600 mg/kg/day	14 days	Dong et al. (2016)
30		Z-VEID-FMK	Caspase-6	Direct	Optic nerve transection, optic nerve crush	Direct inhibition	1,062 RGCs per mm^2^at endpoint compared to 370 RGCs per mm^2^for controls	14 days	Monnier et al. (2011)
31		C6DN (mutant Caspase-6 dominant negative expression construct)	Caspase-6	Direct	Optic nerve crush	Direct inhibition	60% RGC protection with 5 μM treatment	21 days	Vigneswara et al. (2014)
32		VEID-fmk	Caspase-6	Direct	NMDA (glutamate receptor agonist)	Direct inhibition	704 ± 85 cells/mm^2^in treatment group compared to 316 ± 54 cells/mm^2^in control group	31 h	Schuettauf et al. (2011)
33		siCASP6	Caspase-6	Direct	Artery ligation	Direct inhibition	1,084 RGCs per mm^2^for treatment group compared to 662 RGCs per mm^2^for controls	14 days	Shabanzadeh et al. (2015)
34		Z-VEID-FMK	Caspase-6	Direct	Artery ligation	Direct inhibition	1,019 RGCs per mm^2^for treatment group compared to 748 RGCs per mm^2^for controls (34% increase)	14 days	Shabanzadeh et al. (2015)
35		Z-IETD-fmk	Caspase-8	Direct	Anterior chamber microbead injections	Direct inhibition	80% RGC axon survival in the z-IETD-fmk group compared to 66% RGC axon survival in the control group	6 weeks	Yang et al. (2021)
36		Z-IETD-FMK	Caspase-8	Direct	Optic nerve transection, optic nerve crush	Direct inhibition	Significatn increase in RGC density compared to controls	14 days	Monnier et al. (2011)
37		Melatonin	Caspase-8	Indirect	Ocular hypertension injury	Inhibition via suppression of NLRP3 and ASC	Increased RGC density and retinal nerve fiber layer thickness compared to control	ND	Ye et al. (2022)
38		Laquinimod	Caspase-8	Indirect	Ischemia-reperfusion injury	Inhibition via suppression of proinflammatory cytokines IL-1B, TNFα, iNOS, and IL-6	82.92% RGC survival in the LQ group compared to 42.96% survival for controls	7 days	Jiang et al. (2020)
39		IETD-fmk	Caspase-8	Direct	NMDA (glutamate receptor agonist)	Direct inhibition	449 ± 63 cells/mm^2^in treatment group compared to 316 ± 54 cells/mm^2^in control group	31 h	Schuettauf et al. (2011)
40		siCASP8	Caspase-8	Direct	Artery ligation	Direct inhibition	1,090 RGCs per mm^2^for treatment group compared to 662 RGCs per mm^2^for controls	14 days	Shabanzadeh et al. (2015)
41		Z-IETD-FMK	Caspase-8	Direct	Artery ligation	Direct inhibition	931 RGCs per mm^2^for treatment group compared to 748 RGCs per mm^2^for controls (24% increase)	14 days	Shabanzadeh et al. (2015)
42		IETD-CHO	Caspase-8	Direct	Optic nerve transection	Direct inhibition	729 RGCs per mm^2^for treatment group compared to 397 RGCs per mm^2^for controls	14 days	Weishaupt et al. (2003)
43		Cobra venom factor (complement depletion)	Caspase-9	Indirect	Laser photocoagulation	Inhibition via decreased complement activation, MAC deposition, and GFAP expression	41.5% RGC survival in CVF group compared to 28.42% survival for controls	42 days	Jha et al. (2011)
44		Ac-LEHD-CHO	Caspase-9	Direct	Optic nerve transection	Direct inhibition	Increased cell density of 656 RGCs per mm^2^compared to controls	14 days	Kermer et al. (2000)
45		z-LEHD-fmk	Caspase-9	Direct	Optic nerve transection	Direct inhibition	Increased cell density of 714 RGCs per mm^2^compared to controls	14 days	Kermer et al. (2000)
46		Curcumin	Caspase-9	Indirect	Optic nerve cut	Inhibition via reduction in JNK, c-Jun, and ERK signaling	Increased IPL and ONL thickness in treatment group as compared to control	24 h	Buccarello et al. (2021)
47		Erythropoietin	Caspase-9	Indirect	NDMA-mediated excitotoxic retinal damage	Inhibition via binding to RGC erythropoietin receptor to suppress apoptotic signal transduction pathway	84.09% RGC survival rate for treatment group compared to 57.8% for control	7 days	Cheng et al. (2020)
48		LEHD-fmk	Caspase-9	Direct	NMDA (glutamate receptor agonist)	Direct inhibition	211 ± 31 cells/mm^2^in treatment group compared to 633 ± 152 cells/mm^2^in control group	31 h	Schuettauf et al. (2011)
49		Valproate	Caspase-12	Indirect	Ischemia-reperfusion injury	Inhibition by increased GRP78 expression, histone H3 acetylation and decreased CHOP expression	1,620 RGCs per mm^2^for treatment group compared to 916 RGCs per mm^2^for controls	7 days	Zhang et al. (2011)
50		Valproate	Caspase-12	Indirect	Optic nerve crush	Inhibition by suppression of initiation factor 2a-C/EBP homologous protein signaling	Increased RGC density and whole retinal thickness in treatment group compared to control	7 days	Pan et al. (2023)

### Caspase-1

Caspase-1 is atypical for its involvement in inflammation, immune response, and pyroptosis, rather than apoptosis like most other caspases involved in RGC degeneration. While apoptosis is regarded as the key pathway in RGC death, there is potential for a pyroptosis-induced pathway as well. Essential proteins involved in pyroptosis, including GSDM, GSDMDp32, Caspase-1, and Caspase-1p20, are upregulated following increased IOP (Zhang et al., 2021a). Eth D-III positive neurons, indicative of pyroptotic cell death, are significantly amplified following an increase in IOP. Caspase-1 expression is directly correlated with RGC death, increasing in the ganglion cell and inner nuclear layers as soon as 1-h post-retinal ischemia injury (Zheng et al., 2004; Zhang et al., 2019). TUNEL-positive cells indicating RGC death are visible beginning at 3-hour post-retinal ischemia injury (Zheng et al., 2004). Caspase-1 immunoreactivity thus precedes TUNEL activation, indicating that caspase-1 is likely an early activator of neuronal apoptosis. Following the caspase-8-HIF pathway, NRLP12, NLRP3, and NLRC4 activate caspase-1, resulting in IL-1B maturation, neuroinflammation, and pyroptosis (Chen et al., 2020). While caspase-1 expression is affected by inhibitors with upstream targets such as NLRP3 and P2X7R (Zhang et al., 2019), to date there are no successful neuroprotective methods that directly target caspase-1, likely due to the reduced influence of pyroptosis on RGC death, as compared to apoptosis.

### Caspase-2

Caspase-2, unique for its ability to function as both an initiator and executioner caspase, has a recently discovered significance in RGC apoptosis. Several reported pathways, including three-repeat tau production and BH3-interacting domain promotion, utilize caspase-2 as a downstream recipient of upregulation; each differential pathway leveraging caspase-2 cleavage leads to subsequent RGC death (Uchibayashi et al., 2011; Ngolab et al., 2021). Caspase-2 mRNA is detected considerably higher in the RGC layer as compared to other cell types within the retina (Ahmed et al., 2011), but caspase-2 protein presence remains low under conventional circumstances. Following a trauma such as optic nerve crush (ONC) or the more aggressive optic nerve transection (ONT), the active, cleaved form of caspase-2 is detectable as soon as 1-day post-trauma. Active caspase-2 presence correlates closely with apoptotic (TUNEL +) cells and is observable in 90% of apoptotic RGCs, emphasizing both the aggregation and cleavage pathways of caspase-2-mediated apoptosis (Thomas et al., 2018).

### Caspase-3

As a key executioner, caspase-3 proteolytically cleaves a variety of cellular proteins, including PARP, ICAD, Bcl-2, and ROCK-I. The downstream effects directly induce apoptosis, morphologically visualized as cell shrinkage, nucleic acid fragmentation, and cell membrane blebbing (Zalewska et al., 2008). Caspase-3 involvement in RGC death has been continually observed, with protein levels increasing by more than 10-fold following IOP increase or optic nerve injury (Levkovitch-Verbin et al., 2007; Husain et al., 2012). The increase in caspase-3 expression correlates with the respective activity of other pro-apoptotic domains, including MAPK and P-SAPK/JNK, and pro-survival pathways such as PI-3 Kinase, AKT, and p-ERK. The abundance of evidence for caspase-3’s role in RGC apoptotic death renders it an attractive candidate for targeted treatment. Investigation into the role of caspases in RGC apoptosis via complete genetic knockout of the caspase proteins is broadly unfeasible, as observed by hyperplasia and neuronal disorganization in caspase-3 deficient mice that ultimately die at 1–3 weeks of age (Kuida et al., 1996). This is likely due to the universal prominence of caspases in development and neuronal pruning.

### Caspase-6

During apoptosis, caspase-6 localizes in the nucleus, cleaving the nuclear mitotic apparatus (NuMA) as well as structural proteins such as lamin A and alpha-spectrin. As an effector, caspase-6 is especially responsible for the disruption of an apoptotic cell’s structural integrity and dismantling of the cytoskeleton (Wang et al., 2015). Substrates of caspase-6 include transcription factors such as nuclear factor κB (NF-κB), special AT-rich sequence-binding protein 1 (SATB1), activating protein 2α (AP-2α), and CREB-binding protein (CBP) (Levkau et al., 1999). The presence of the active, cleaved caspase-6 p10 subunit in RGCs is significantly increased 4 days post-retinal injury. Caspase-6 also shows higher immunostaining reactivity in the ganglion cell layer and nerve fiber layer following injury, clearly demonstrating its role in retinal nerve apoptosis (Monnier et al., 2011).

### Caspase-7

An efficient executioner protein, caspase-7 is activated by caspase-8 or caspase-9 through proteolytic cleavage following death receptor engagement. While there is an overlap between the downstream recipients of caspase-3 and caspase-7 executioner activity, caspase-7 has the sole responsibility for the cleavage of several substrates in the apoptotic cascade, including kinectin and co-chaperone P23 (Machleidt et al., 1998; Boucher et al., 2012). Caspase-3 involvement in RGC apoptosis is far more reported than its counterpart caspase-7, but caspase-7’s role in injury-induced RGC death has also been observed.

The global knockout of caspase proteins in animal models is often unfeasible due to the ubiquitous presence of caspases throughout developmental pathways. A notable exception to this trend is caspase-7, given recent evidence that caspase-7 knockout mice can be developed and remain viable (Choudhury et al., 2015). Caspase-7 knockout mice were protected against RGC death, demonstrated by 2x higher RGC density and a significant reduction in retinal layer thinning. In addition to the traditional intrinsic activation pathway, caspase-7-mediated activation of the RGC apoptotic cascade is also induced by calpain-1, a prevalent enzyme involved in cell signaling and cytoskeletal dynamics (Choudhury et al., 2015). Given the evidence of caspase-7’s role in RGC death as shown by caspase-7^−/−^ mice, strategies to inhibit caspase-7 serve as an attractive avenue for preventing RGC apoptosis.

### Caspase-8

Caspase-8’s recruitment to the death-inducing signaling complex, and subsequent activation of downstream caspases, establishes its prominence as one of the key initiators of apoptosis (Kuwana et al., 1998). Glaucoma cases with normal range IOP are often associated with unstable hemodynamic characteristics and can be modeled by induction of systemic hypotension. Increased angiotensin II is observed in this model, in addition to upregulation of GFAP, Iba-1, TNF-α, RIP3, and an increase in the ratio between active and inactive caspase 8 (Jeon et al., 2022). Caspase-8 is involved in the pathways of TLR4-facilitated IL-1β production and NLRP1/NLRP3 inflammasome activation, both of which underlie the mechanism of RGC death. This signal transduction can transpire *via* both a caspase-1-dependent and caspase-1-independent pathway (Chi et al., 2014). IL-1β production is additionally influenced by HMGB1 elevation and the NF-kβ pathway, both of which are also affected by caspase-8 under high IOP circumstances (Chi et al., 2015). It follows that mRNA levels of IL-1β and TNF-α decrease significantly following treatment of caspase-8 inhibitor (Jiang et al., 2020). Caspase-8 activation following retinal injury has been continually observed, with the increase in caspase-8 mRNA and protein expression visible as soon as 6-h post-injury (Chi et al., 2014; Grotegut et al., 2021).

The role of caspase-8 in the glaucomatous eye was recently elucidated in a dual approach study, utilizing both a caspase-8 inhibiting pharmacological treatment and caspase-8 astroglia genetic deletion (Yang et al., 2021). Despite the difficulty of completely knocking out caspase proteins for the purpose of experimental assessment, astroglia-specific caspase-8 knockout mice are viable. When given a microbead injection to induce axon loss, astroglial caspase-8 knockout mice demonstrated a 30% increase in RGC survival, suggesting the fundamental role of caspase-8 in neurodegeneration (Yang et al., 2021). Of note, necroptosis appears in caspase-8 knockout mouse astroglia that is absent in normal mice treated with caspase inhibitors.

### Caspase-9

As a key component of the intrinsic apoptosis pathway, caspase-9 is activated by Bax/Bcl-2-mediated transfer of cytochrome-c into the cytosol. Cytochrome-c association with Apaf-1 and dATP forms the apoptosome, recruiting pro-caspase-9 and inducing its activation through proteolytic cleavage (Elmore, 2007). There is a clear association between elevated IOP, RGC death, and cleaved caspase-9 levels, demonstrating a caspase-9-dependent pathway of intrinsic RGC apoptosis. Cleaved caspase-9 is absent in control retinas but present in the ganglion cell layer and in the retinas of rats with IOP above 32 mmHg and 35 mmHg respectively (Hänninen et al., 2002). Caspase-9 activation in the retina increases three-fold following trauma such as optic nerve transection (Kermer et al., 2000). No caspase-9 genetic knockout models exist to date, as caspase-9^−/−^ mice die perinatally, exhibiting cerebral enlargement and excessive neuronal growth from lack of apoptosis (Kuida et al., 1998).

## Therapeutics

### Caspase-targeted siRNAs

There exist several novel neuroprotective strategies founded on the direct inhibition of caspases by chemically modified siRNAs that prevent synthesis of the target protein. Specific siRNAs have demonstrated significant success in animal models at inhibiting a variety of both initiator and effector caspase targets, including caspase-2, caspase-3, caspase-6, and caspase-8. siRNAs are thus one of the most explored neuroprotective treatments in the clinical domain.

Multiple groups have reported caspase-2 suppression by such siRNAs (siCASP2s) (Ahmed et al., 2011; Thomas et al., 2018; Solano et al., 2014). siCASP2 preserves up to 98% of RGCs as compared to controls in ONC models, providing sustained neuroprotection for up to 30 days when given as a single intravitreal injection (Ahmed et al., 2011). Similar neuroprotection is observed in an ONT model, where RGC density is 2.5 times greater 2 weeks post-siCASP2 treatment (Ahmed et al., 2011). The same group saw similar neuroprotection of RGCs in a closed globe blunt ocular trauma model of photoreceptor and RGC death. siCASP2 treatment retains up to 46.5 µM BRN3A + cells per 1,000 µM of retinae, a measure of RGC survival, as compared to 39.9 BRN3A + cells in injured controls (Thomas et al., 2018). This caspase-2 targeting siRNA is now ongoing Phase II/III clinical trial as a fully developed pharmaceutical under the identifier QPI-1007 (Solano et al., 2014).

Caspase-3 inhibition is a heavily explored avenue of neuroprotection, with numerous inhibition methods currently in existence and others in development (Tawfik et al., 2021; Ishikawa et al., 2012; Sánchez-Migallón et al., 2016; Chen et al., 2001). Caspase-3 is the most common target of inhibition in caspase-related RGC apoptosis investigation. Caspase-3 specific siRNA encapsulated in polybutylcyanoacrylate nanoparticles (CaspNPs) are a promising avenue of neuroprotection due to their sustained ocular longevity and targeted release when delivered intraocularly (Tawfik et al., 2021; Ishikawa et al., 2012). CaspNP treatment reduces the integrated optical density (IOD) of caspase-3 immunofluorescent signal intensity by more than three times, indicating the nanoparticles’ strong inhibition capability. Rats treated with CaspNPs show 17% increase in RGC survival with the most statistical significance in the chronic stage, post-day 21 of treatment (Tawfik et al., 2021). An alternative production of caspase-3 siRNA inhibition shows similar neuroprotection following ischemia-reperfusion injury. Intraocular injection of caspase-3 siRNA reduces the expression of caspase-3 mRNA, retains retinal thickness, and increases the survival rate of RGCs as compared to the saline control group (Ishikawa et al., 2012).

Following uniform retinal ischemic injury by ophthalmic artery ligation, caspase-6-targeted siRNA injected intravitreally retains 1,084 RGCs/mm^2^ compared to 662 RGCs/mm^2^ for controls, demonstrating considerable neuroprotection (Shabanzadeh et al., 2015). An alternative siRNA to caspase-6 (C6DN) linked to a cell penetrating peptide Penetratin1 (Pen1) also displays stabilizing effects on the RGC layer. Following optic nerve crush, C6DN-Pen1 causes a dose-dependent increase in surviving RGCs by up to 60% protection at maximum levels. These effects are also observed when delivered in tandem with a chemically stabilized siRNA to caspase-2 (Vigneswara et al., 2014). Caspase-6 is thus a fundamental effector in the RGC apoptosis pathway and mediates the actions of other family members such as caspase-2 and caspase-8 (Monnier et al., 2011; Shabanzadeh et al., 2015; Vigneswara et al., 2014).

Two variants of caspase-8-targeted siRNA (CASP8 siRNA) exist to date, with both compounds displaying strong neuroprotective capabilities (Shabanzadeh et al., 2015). Upon retinal ischemic injury by ophthalmic artery ligation, intravitreal injection of CASP8 siRNA 1 and CASP8 siRNA2 increases RGC survival by an average of 60%, with CASP8 siRNA1 retaining 1,090 RGCs/mm^2^ and CASP8 siRNA 2 retaining 1,044 RGCs/mm^2^, compared to 662 RGCs/mm^2^ for controls.

### Peptidomimetic inhibitors

Peptidomimetic inhibitors mimic a caspase-cleaving substrate using specific amino acid sequences such as YVAD, DEVD, VDVAD, IETD, VEID, and LEHD (Monnier et al., 2011; Kermer et al., 2000; Sánchez-Migallón et al., 2016; Shabanzadeh et al., 2015; Schuettauf et al., 2011; Weishaupt et al., 2003). N-terminal blocking groups stabilize peptide inhibitors, allowing them to bind to caspase active sites with chemical groups such as fluoromethylketone (fmk) and chloromethylketone (cho) (Chen et al., 2001; Weishaupt et al., 2003). Synthetic peptide-based inhibitors are thus another attractive candidate for targeted caspase inhibition. z-VDVAD-fmk is a synthetic peptide-based inhibitor targeted for caspase-2. Following neurotrauma from ONC, z-VDVAD-fmk protects RGCs from death by up to 60% 15 days post-trauma. This neuroprotection is mechanistically supported by suppression of cleaved caspase-2 (C-CASP2), demonstrated by an 85% reduction in C-CASP2 levels post-ONC compared to controls (Vigneswara et al., 2012). It is important to note that, like many alternative caspase inhibition methods, z-VDVAD-fmk promotes neuron conservation, but not axon regeneration.

z-DEVD-fmk is a synthetic peptide-based inhibitor that targets caspase-3 irreversibly. Following optic injury, z-DEVD-fmk treatment preserves the RGC population and decreases caspase-3 expression in both rabbits and mice (Sánchez-Migallón et al., 2016). DEVD-CHO, a tetrapeptide caspase-3 inhibitor, is an alternative to z-DEVD-fmk that contains the same “DEVD” recognition sequence for caspase-3; however, DEVD-CHO works in a reversible mechanism and contains an additional stability carbonyl group, unlike its counterpart. DEVD-CHO promotes RGC survival in a dose-dependent manner, doubling the survival rate of RGCs exposed to glutamate excitotoxicity (Chen et al., 2001). Of important note, the neuroprotective effects of peptidomimetic caspase-3 inhibitors are only observed when delivered immediately following optic nerve injury (Sánchez-Migallón et al., 2016). The ephemeral benefits granted by these peptide inhibitors could potentially be less applicable to the chronic degenerative state often seen in glaucoma.

z-VEID-fmk, a peptidomimetic inhibitor targeted for caspase-6, demonstrates strong neuroprotective ability in an ischemic model. Following optic nerve axotomy, z-VEID-fmk reduces RGC death by a factor of 2.2, also improving intraretinal axon integrity as demonstrated by thicker axon bundles throughout the retina (Monnier et al., 2011; Shabanzadeh et al., 2015). Of note, caspase-6 inhibition with z-VEID-fmk provides one of the few documented examples of axonal regeneration following optic nerve injury. z-VEID-fmk also induces axonal outgrowth of myelin and is unique in its ability to regenerate axons up to eightfold and beyond the original lesion site (Monnier et al., 2011). Following excitotoxic RGC stimulation by N-methyl-D-aspartate (NMDA), inhibition by z-VEID-fmk induces 41.6% RGC survival compared to 18% RGC survival for controls (Schuettauf et al., 2011). *In vivo*, caspase-6 can similarly be inhibited by z-VEID-fmk as well as small molecule inhibitor SIMA 13a, with both treatments significantly retaining RGC density following axotomy by two to three-fold (Monnier et al., 2011).

z-IETD-fmk, a synthetic peptide-based caspase-8 inhibitor, affords 40% RGC protection following IOP increase, as assessed by optic nerve axon counts and pattern electroretinogram (PERG) responses (Yang et al., 2021). z-IETD-fmk preserves RGCs following optic nerve transection, artery ligation, and excitotoxic stimulation by NMDA, increasing RGC density by 24%, 24%, and 33% respectively (Shabanzadeh et al., 2015; Schuettauf et al., 2011). IETD-CHO caspase-8 inhibitor targets the same amino acid sequence as z-IETD-fmk but is further stabilized by a carboxylic acid anhydride group. IETD-CHO treatment protects RGC density even further, retaining 729 RGCs/mm2 as compared to 330 RGCs/mm2 for controls and 694 RGCs/mm2 when treated with z-IETD-fmk following optic nerve transection (Weishaupt et al., 2003).

Irreversible caspase-9 inhibitor z-LEHD-fmk increases RGC density by 714 cells per mm^2^ as compared to controls. Ac-LEHD-CHO targets the same amino acid structure as z-LEHD-fmk, but the addition of the carboxylic acid anhydride group renders it a more stable and reversible inhibitor of caspase-9. Ac-LEHD-CHO significantly preserves RGCs following optic nerve trauma, increasing cell density by 656 cells per mm^2^ as compared to controls. Of note, non-caspase-9 selective inhibitors such as caspase-3-specific z-DEVD-cmk, also display caspase-9 reducing ability (Kermer et al., 2000). This is conceivable given the DEVD inhibitor group’s affinity for not only its target of group III caspases (caspase −2, −3, −7), but for group II caspases (caspase −6, −8, −9, 10) as well (Garcia-Calvo et al., 1998).

### Neurotrophic factors

Given the ubiquitous involvement of caspases in various apoptotic pathways, it follows that caspase expression is also affected by inhibitors with upstream targets. Caspases are frequent targets of indirect inhibitors that often have multiple targets but can be especially efficient at inhibition of caspase; in some cases, these indirect inhibitors can protect RGCs even more than direct peptidomimetic or siRNA inhibitors of caspase (Sánchez-Migallón et al., 2016).

Neurotrophic factors are a clear choice of indirect caspase inhibitor, given the canonical pathway linking IOP elevation, neurotrophic factor reduction, and the onset of optic neuropathy (He et al., 2018; Gupta et al., 2014; Klöcker et al., 1997). Neurotrophic factors with the most retinal neuroprotective potential include BDNF, PEDF, and IGF-I (Vigneswara and Ahmed, 2019; Jeon et al., 2022; Kermer et al., 2000; Kurokawa et al., 1999). BDNF exhibits a clear neuroprotective effect by targeting caspase-2; following retinal ischemic-reperfusion injury, treatment with BDNF decreased caspase-2 production and significantly retained RGC density by 36.7% (Kurokawa et al., 1999). BDNF also inhibits caspase-3, inducing neuroprotection almost identical to z-DEVD-fmk in mice RGCs (Jeon et al., 2022). Neurotrophin PEDF delivered intravitreally suppresses caspase-2 mRNA by a factor of 1.85 and increases RGC survival by 63.2%. Comparable prognoses are observed in eye drop delivery of PEDF-34, with caspase-2 mRNA suppression by a factor of 3.04% and 81.9% increase in RGC survival (Vigneswara and Ahmed, 2019). Neurotrophic treatment also appears to block caspase-9 apoptotic activity, as intraocular injection of growth factors IGF-I and BDNF both preserve the RGC layer and reduce caspase-9 activation following neurotrauma (Kermer et al., 2000). This supports the theory of neurotrophic factor-induced neuroprotection by obstruction of the intrinsic mitochondrial apoptotic pathway.

### Anti-inflammatories

Modulation of the inflammatory process, namely, suppression of cytokine action and modification of macrophage response, is a potential pathway for the mitigation of RGC death (Zhang et al., 2005). Several anti-inflammatory compounds have been identified to inhibit various caspases and simultaneously protect RGCs following retinal damage. Curcumin and gingko biloba extract, two natural compounds with antioxidant and anti-inflammatory properties, demonstrate caspase-9-related ability to disrupt RGC apoptosis. Gingko biloba extract reduces hydrogen peroxide-induced apoptosis through reduction in Bax/Bcl-2 and caspase-9 expression (Yu et al., 2022). Similarly, curcumin prevents apoptosis following optic nerve injury through a decrease in MAPK family members such as p-ERK, p-JNK, and p-c-Jun, eventually reducing caspase-3 and caspase-9 expression (Buccarello et al., 2021). Kaempferol, a flavonoid compound known for its anti-inflammatory properties, recently demonstrated additional RGC neuroprotective characteristics. Following retinal I/R injury, kaempferol treatment significantly decreases expression of caspase-3, increases retinal thickness by over 10%, and increases RGC survival by over 20%. Kaempferol’s neuroprotective mechanisms also inhibit caspase-8 and NLRP/NLRP3 inflammasomes, mediated through reduction in the JNK and upregulation of the NF-kB pathways (Lin et al., 2019). Resveratrol, a polyphenol antioxidant, demonstrated multifaceted RGC neuroprotection following ischemia-reperfusion retinal injury. Intraperitoneal injection of resveratrol preserves RGCs, retains retinal thickness, and downregulates caspase-3 expression (Ji et al., 2021). Resveratrol and kaempferol both induce similar downregulation in caspase-3 and caspase-8 expression, but not in other caspases of interest such as −1, −6, −7, or −9 (Lin et al., 2019; Ji et al., 2021; Seong et al., 2017).

### Hormonal treatments

A wide range of endogenous hormones have observed inhibitive properties on various caspases. Cytoprotectant hormone estrogen displays protection of optic nerve head astrocytes (ONHAs) through reduction of the caspase-3 mediated tau cleavage pathway. Estrogen treatment significantly reduces caspase-3 activation and tau truncation in ONHAs with tert-butyl hydroperoxide-induced oxidative stress (Means et al., 2021). Erythropoietin, an abundant glycoprotein hormone, displays similar retinal neuroprotective effects through downregulation of upstream indicator calpain, Bax, and caspase-9 sequentially. In rats with NMDA-induced excitotoxic retinal damage, erythropoietin significantly preserves RGC density, reduces calpain-positive RGCs after 6 h, and subsequently reduces caspase-9-positive RGCs after 42 h (Cheng et al., 2020). L-817,818, agonist for alternate peptide hormone somatostatin, displays equivalent capacity to downregulate caspase-3 and caspase-9 expression following ocular hypertension induction, subsequently attenuating RGC death (Zhang et al., 2021b). Melatonin also exhibits a neuroprotective effect via reduction of cleaved caspase-3 and caspase-8, retaining retinal nerve fiber layer thickness and increasing RGC counts following acute ocular hypertension injury. This is attained through decreased activation of MLKL, RIP1, RIP3 and inhibited expression of NLPR3, ASC, and IL-1β, as seen in multiple studies (Zhang et al., 2021a; Ye et al., 2022).

### Immunomodulators and cytoprotectants

Immune system involvement in glaucoma development is increasingly hypothesized (Bell et al., 2018) and compounds with both immunomodulating and immunosuppressing capability have demonstrated caspase-inhibiting and RGC-protecting abilities. The trifold complement system is an important immune component; in rats with increased IOP, increased complement activation induces membrane attack complex accumulation, resulting in both intrinsic and extrinsic pathways of RGC apoptosis. Depletion of the complement system by cobra venom factor reduces TUNEL+ apoptotic RGCs and decreases both caspase-8 and caspase-9 expression (Jha et al., 2011). Cyclosporine A is a fungus-derived immunosuppressant that displays neuroprotective ability, increasing RGC viability by 2.5 times in cells with glutamate-induced excitotoxity (Schultheiss et al., 2014). RGC viability directly correlates with caspase-3 and caspase-7 activity, which are reduced by 1.3-fold following cyclosporine A treatment. Laquinimod, an immunomodulatory drug, reduces production of cleaved caspase-8 and its downstream signaling molecules, inhibiting neuronal apoptosis caused by dysregulated neuroinflammation (Jiang et al., 2020).

Spontaneous RGC death independent of IOP is observed in a normal-tension glaucoma model simulated by Glutamate Aspartate Transporter (GLAST)^+/−^ mice. GLAST^+/−^ mice are a valuable tool for the investigation of retinal disease, particularly those involving excitotoxicity, as GLAST plays a crucial role in the regulation of the neurotransmitter glutamate in the retina. Administration of geranylgeranylacetone, a chaperone inducer of HSP70, suppresses RGC death and reduces caspase-3 and caspase-9 activity in GLAST^+/−^ mice (Dong et al., 2016). Necrostatin-1, another cytoprotectant with neuroprotective ability, uniquely inhibits caspase-1 and the pyroptosis pathway. Suppression of the RIP1/RIP3, MLKL pathway by GSK872 and Necrostatin-1 downregulates caspase-1 and IL-1β expression, reducing RGC death (Liu et al., 2022).

### Biochemical inhibitors and analogues

Latanoprost, a prostaglandin F2a analog, is a widely popular anti-glaucoma medication utilized for IOP reduction. Latanoprost reduces both activated caspase-3 immunoreactive cells and TUNEL-positive apoptotic retinal cells, suggesting that latanoprost has neuroprotective effects independent of its IOP-reducing mechanisms (Nakanishi et al., 2006; Kanamori et al., 2009). This cytoprotective capability is observed in a dose-dependent manner and relies on the MAPK pathway. Morphine, a common and potent analgesic, induces opioid receptor activation in RGCs to suppress neuro-destruction (Husain et al., 2012). Following saline-induced IOP increase, treatment of morphine reduces caspase-3, caspase-8, and TNF- α expression. This neuroprotective effect of morphine is observed through significantly increased RGC counts and recovery of retinal PERG amplitudes in treatment eyes as compared to controls. Sildenafil citrate, a phosphodiesterase type 5 (PDE5) inhibitor, targets caspase-7 and reduces its upregulation post ischemia-reperfusion. Sildenafil citrate preserves the RGC layer and increases RGC cell count as compared to controls, demonstrating a neuroprotective effect of sildenafil citrate on caspase-7-related apoptosis, but the same result is not seen in caspase-6 or caspase-9 (Zanoni et al., 2017).

## Discussion

Caspase proteins are a fundamental aspect of the apoptotic process, RGC death, and the progression of degenerative eye diseases such as glaucoma. While elevated IOP is the main risk factor in ocular nerve degeneration, it is not ubiquitous in its extent. There very likely exist other pathologic mechanisms behind glaucoma progression and accordingly, alternative methods of treatment. It is well understood that RGC death lies at the core of neurogenerative eye diseases, rendering the minimization of RGC loss a strong prospect for future treatment (Schuster et al., 2020; Vishwaraj et al., 2022). RGCs lack the ability to divide or regenerate, reinforcing the irreversibility of their death and the importance of their preservation. Inhibition of caspases to prevent RGC death is an especially promising avenue of optic neuroprotection and possible through mediation of one of several upstream pathways that affect caspase activation. Suppression of pro-apoptotic axes such as NLRP1/NLRP3, ASC, IL-1β, TNF-α and MAPKs reduces both initiator and effector caspase activity (Levkovitch-Verbin, 2015; Almasieh et al., 2011; Zhang et al., 2019; Chen et al., 2020). Similarly, upregulation of anti-apoptotic axes such as Bcl-2, Pi3K/Akt, and NF-kβ accomplishes an identical caspase-inhibiting effect (Kuwana et al., 1998; Zalewska et al., 2008). Direct caspase inhibition by synthetic peptides allows for targeted obstruction of specific caspases at various intervals of the apoptotic cascade. Peptidomimetic inhibitors mimic a substrate that the caspase would naturally cleave using specific amino acid sequences such as YVAD (caspase-1), DEVD (caspase-3), VDVAD (caspase-2), IETD (caspase-8), VEID (caspase-6), and LEHD (caspase-9) (Monnier et al., 2011; Kermer et al., 2000; Sánchez-Migallón et al., 2016; Schuettauf et al., 2011). Direct caspase inhibition can also be achieved through caspase-specific siRNAs that prevent synthesis of the target protein. Specific siRNAs have been developed for a variety of both initiator and effector targets, including caspase-2, caspase-3, caspase-6, and caspase-8 (Thomas et al., 2018; Solano et al., 2014; Shabanzadeh et al., 2015; Vigneswara et al., 2014). siRNAs have demonstrated significant success in animal models and are the most explored neuroprotective treatment in the clinical domain. Both methods of direct inhibition–siRNA and synthetic peptides–have proved especially effective at RGC preservation and likely have more potential for clinical application, given their higher efficacy and lower potential for unintended extracellular effects.

As inhibiting methods continue to be developed, the absolute clinical end points indicative of neuroprotective outcomes could require reassessment; the measurements of glaucomatous protection precedingly used in clinical trials focus more on evaluating overall visual field acuity rather than RGC preservation. Since RGC preservation is currently the primary metric for assessing the potential of caspase-inhibiting therapies, it is critical to determine whether this measure actually correlates with improvements in visual acuity as candidates advance from development to clinical evaluation. Directly comparing these two metrics is challenging: animal models cannot accurately report visual acuity, while human studies cannot quantify RGCs through histology and must instead rely on optical coherence tomography (OCT)-based estimations.

In mouse models, visual acuity is measured by behavioral evaluation through virtual reality optomotor systems and optical imaging of intrinsic signals (Krempler et al., 2011; Torero et al., 2011). Decreases in RGCs following induced retinal ischemia, as measured through histological analysis, are also correlated with a decline in visual acuity within these animal models. Treatments such as simvastatin, which have been shown to support RGC survival, also appear to be associated with notable improvements in visual acuity (Krempler et al., 2011). In Rhesus monkeys, quantification of RGC density through histology show decreases that correlate with visual field defects measured by behavioral testing perimetry when compared with a log-log model (Harwerth et al., 2004). In human observational cohort studies, glaucoma patients with reduced RGCs, as measured by OCT and automated perimetry, exhibit a corresponding decline in visual field acuity, as assessed by the Visual Field Index (Medeiros et al., 2012). Similarly, studies of homonymous visual field defects show a correlation between RGC loss measured by OCT and visual field reduction determined by automated Humphrey field testing (Schneider et al., 2019). Collectively, both human studies and animal models effectively demonstrate a correlation between RGC survival and preserved visual acuity. In the future development of caspase inhibitors, it will be crucial to broaden the focus beyond RGC survival and directly assess improvements in visual acuity, ideally utilizing a combination of both human studies and animal behavior models. Previous clinical trials evaluating neuroprotection in the glaucomatous eye, including the use of oral memantine and nicotinamide, assess measures such as visual field testing and optic nerve damage (Weinreb et al., 2018; De Moraes et al., 2022). In addition to these primary efficacy metrics, the evaluation of RGC loss through OCT retinal thickness measurement, electroretinography (ERG), or fluorescein angiography could provide more direct evidence of the RGC-retaining potential of new treatments. Trials for other disorders possessing a clear association with RGC loss, particularly optic neuritis, could also serve as validation for future therapeutic success in neuroprotection (Andorrà et al., 2020). Assessing RGC axonal survival offers another important metric, as RGC preservation does not necessarily equate to maintained visual function if axonal degeneration is present (Krauss et al., 2020). The gene *Sarm1* plays a key role in axonal degeneration and was recently implicated in RGC loss in glaucoma (Zeng et al., 2024). *Sarm1* presents a promising target for future studies on RGC axon survival, its contribution to neuroprotection, and associated improvement in visual acuity.

The four proposed criteria for assessing the neuroprotective potential of pharmaceutical agents (Levkovitch-Verbin et al., 2007) could assist in bridging this gap between translational evidence in RGC survival and clinical improvement of visual acuity. Criteria 3) and 4) have proven to be the most challenging specifications for neuroprotective candidates to fulfill. Criteria 3 specifies that candidates should reach neuroprotective concentrations in the posterior segment after clinical dosing, while criteria 4 mandates the observation of neuroprotection in clinical trials. Conventional routes of drug administration, including intraocular injection and topical application, are frequently unsuccessful at retaining sufficient posterior segment concentrations following treatment due to the structurally defensive nature of ocular anatomy. Impediments to sustained drug delivery in the eye include the heightened permeability of the corneal and retinal pigment epitheliums, vitreous chamber diffusion, and lacrimal duct outflow (Nayak and Misra, 2018). Therefore, the greatest challenge lies not in the creation of pharmaceutical agents that satisfy the first two neuroprotective agent assessment criteria (containing target receptors in the retina or optic nerve and demonstrating significant improvement to RGC survival), but rather targeting their delivery for sustained and focused intraocular release. Directed routes of administration, including subconjunctival, subtenon, posterior juxtascleral, retrobulbar, and peribulbar, and engineered delivery platforms, such as intravitreal implants, microspheres, nanoparticles, and liposomes, are thus being explored to establish a more efficient therapeutic with fewer off-target effects (Varela-Fernández et al., 2020). As these systems continue to develop, it will be important to evaluate whether they not only enhance traditional outcomes like RGC survival but also lead to meaningful improvements in visual acuity.

It has been clearly demonstrated that caspase-inhibiting neuroprotective treatments have a significant ability to reduce RGC death in a variety of retinal injury models. Further study into caspase-inhibiting methods, treatments, and delivery systems has the potential to afford ubiquitous neuroprotection to the glaucomatous eye.

## Glossary

RGC: Retinal ganglion cell
IOP: Intraocular pressure
C: D: Cup-to-disc
GPCR: G-protein-coupled receptor
BDNF: Brain-derived neurotrophic factor
TrkB: Tropomyosin receptor kinase B
p75NTR: P75 neurotrophic receptor
CNTF: Ciliary neurotrophic factor
PEDF: Pigment epithelium-derived factor
GNDF: Glial cell line-derived neurotrophic factor
PI3K: phosphatidylinositol 3-kinase
Akt: protein kinase B
MAPK: mitogen-activated protein kinase
ERK: Extracellular signal-regulated kinases
JNK: Jun N-terminal kinases
TNF: tumor necrosis factor
Fas-L: Fas ligand
TRAIL: TNF-related apoptosis-inducing ligand
TNFR: TNF receptor
FADD: Fas-associated protein with death domain
TRADD: Tumor Necrosis Factor Receptor-1-Associated Death Domain
DISC: death-induced signaling complex
BID: Bcl2 Interacting Protein
MOMP: Mitochondrial outer membrane permeabilization
Smac: Second mitochondria-derived activator of caspase
DIABLO: Direct inhibitor of apoptosis-binding protein with low pI
Apaf-1: Apoptotic protease activating factor 1
ONC: Optic nerve crush
ONT: Optic nerve transection
NuMA: Nuclear mitotic apparatus
NF-kB: Nuclear factor κB
SATB1: Special AT-rich sequence-binding protein 1
AP-2α: Activating protein 2α
CBP: CREB-binding protein
siCASP2: Caspase-2 suppressing siRNA
CaspNPs: Caspase-inhibiting nanoparticles
IOD: Integrated optical density
C6DN: Caspase-6 suppressing siRNA
Pen1: Penetratin1
Fmk: Fluoromethylketone
Cho: Chloromethylketone
NMDA: N-methyl-D-aspartate
PERG: Pattern electroretinogram
ONHA: Optic nerve head astrocytes
GLAST: Glutamate Aspartate Transporter
PDE5: Phosphodiesterase type 5
OCT: Optical coherence tomography
ERG: Electroretinography
